# Predictive factors and repetition numbers for intraoperative additional resection of initially involved soft tissue resection margins in oral squamous cell carcinoma: a retrospective study

**DOI:** 10.1186/s12957-023-03192-6

**Published:** 2023-09-27

**Authors:** Mark Ooms, Lisa Ponke, Philipp Winnand, Marius Heitzer, Florian Peters, Tim Steiner, Frank Hölzle, Ali Modabber

**Affiliations:** Department of Oral and Maxillofacial Surgery, RWTH Aachen University, Pauwelsstraße 30, 52074 Aachen, Germany

**Keywords:** Oral squamous cell carcinoma, Soft tissue, Resection margins, Intraoperative additional resection, Predictive factors, Repetition numbers

## Abstract

**Background:**

Intraoperative additional resection (IAR) of initially microscopically involved soft tissue resection margins negatively impacts tumor recurrence in oral squamous cell carcinoma (OSCC). Increasing the selected initial macroscopic resection margin distance beyond the tumor tissue may help prevent IAR; however, the existence of predictive factors for IAR and IAR repetition numbers remains unclear. This study aimed to identify predictive factors for IAR and to evaluate the IAR repetition numbers in soft tissue for surgically treated OSCC.

**Methods:**

A cohort of 197 patients surgically treated for OSCC between 2008 and 2019 was retrospectively reviewed (44 patients with IAR and 153 patients without IAR). Clinical parameters (tumor location, midline involvement, clinical T-status, time between staging imaging and surgery, bone resection, monopolar use, and reconstruction flap size) and histopathological parameters (pathologic T-status [pT-status], grading, vascular invasion, and lymphatic invasion) of the two groups were compared.

**Results:**

Patients with and without IAR differed in their histopathological parameters, such as pT-status above 2 (47.7% vs. 28.1%, *p* = 0.014) and lymphatic invasion (13.6% vs. 4.6%, *p* = 0.033); however, their clinical parameters were similar (all *p* > 0.05). Only pT-status above 2 was predictive for IAR in a multivariable regression analysis (odds ratio 2.062 [confidence interval 1.008–4.221], *p* = 0.048). The IAR repetition numbers varied from zero to two (zero = 84.4%, one = 11.4%, and two = 2.3%).

**Conclusions:**

Only postoperative available pT-status was identified as a predictive factor for IAR, underscoring the importance of improving preoperative or intraoperative tumor visualization in OSCC before selecting the initial macroscopic resection margin distance to avoid IAR.

## Background

Oral squamous cell carcinoma (OSCC) is a common head and neck malignancy that is characterized by high recurrence rates and poor survival despite advances in therapeutic strategies [1, 2].

Surgery is the standard initial treatment for resectable OSCCs, and the aim of surgery is to achieve complete tumor resection with adequate microscopic resection margins (≥ 5 mm) [3–6]. The selected initial macroscopic resection margin distance beyond the tumor tissue is commonly 1 cm, which is a compromise between achieving adequate microscopic resection margins and preserving satisfactory residual function [4, 7, 8]. Intraoperative additional resection (IAR) is performed to address microscopically involved resection margins in soft tissue identified by intraoperative frozen section margin assessment [9, 10]. However, errors in the relocation of the initial margin and the interpretation of frozen sections reduce the reliability of IAR in terms of achieving definite microscopically uninvolved resection margins [11–14]. Indeed, IAR of initially microscopically involved resection margins has been shown to not provide local control equivalent to that in initially microscopically uninvolved resection margins [4, 9, 10, 12, 15].

Therefore, the outcome of surgical treatment of OSCCs could be improved by preventing IAR, possibly by selecting an initial macroscopic resection margin distance beyond the tumor tissue that is greater than the commonly used 1 cm [8, 11, 12, 16]. However, there is currently insufficient information available on the predictive factors for IAR and the IAR repetition numbers, which means it is challenging to assess when and how to increase the initial selected macroscopic resection margin distance beyond the tumor tissue to greater than 1 cm. This situation has arisen, because the few studies available have described rather than primarily investigated the topic, did not consider potentially relevant parameters, such as time between imaging and surgery and the resection and reconstruction methods used, and generally lacked information on the IAR repetition numbers [3, 9].

Therefore, the aim of this study was to identify predictive factors for IAR and evaluate the IAR repetition numbers in soft tissue for surgically treated OSCC.

## Methods

### Study population

This retrospective study was approved by the local ethics committee of the Medical Faculty of RWTH Aachen University (EK 039–20). The local ethics committee of the Medical Faculty of RWTH Aachen University allowed us to waive the requirements for securing informed consent from the participants of this human-based study. All methods were in accordance with the Declaration of Helsinki.

The study population consisted of 197 patients who were treated for primary OSCC in the Department for Oral and Maxillofacial Surgery (Faculty of Medicine, RWTH Aachen University) between 2008 and 2019 according to established guidelines [17]. The following exclusion criteria were applied: non-curative treatment; cancer already distant at the time of diagnosis; history of malignancy in the head or neck region, radiotherapy to the head or neck, and or cancer other than head or neck cancer that had not been treated curatively more than five years previously; neoadjuvant radiotherapy or radiochemotherapy; additional postoperative tumor resection; and incomplete tumor resection. In addition, patients with incomplete data sets were excluded. The data analyzed in this study were obtained from clinical notes, surgical reports, and pathology reports.

Staging was performed for all patients using computer tomography of at least the head and neck region (and additionally of the thoracic and abdominal regions, depending on the stage of disease) according to established guidelines [17]. Surgical tumor resection was performed with a selected initial macroscopic resection margin distance of 1 cm three-dimensionally beyond the tumor tissue. For microscopically involved resection margins determined by frozen section margin assessment of the tumor bed (defined as involved resection margins if invasive carcinoma or carcinoma in situ was present), IAR with a width of 5 mm was performed until microscopically uninvolved resection margins were achieved. To determine the status of the definite resection margins, both the final formalin-fixed resection margins of the tumor specimen and additional resection margins of the tumor bed were combined (defined as uninvolved if no invasive carcinoma or carcinoma in situ was present).

The clinical preoperative parameters assessed were tumor location, tumor midline involvement, clinical T-status (cT-status), and time interval between imaging for staging and surgical therapy. The tumor location was determined clinically and radiologically. Midline involvement was determined clinically and radiologically and defined as positive if the macroscopic tumor specimen involved the midline. The cT-status was determined according to the Union for International Cancer Control (UICC) staging classification (UICC 6th edition [2008–2009], 7th edition [2010–2016], and 8th edition [2017–2019]). The time interval between staging imaging and surgical therapy was determined as the time between the computer tomography of the head and neck and the day of surgery. The clinical intraoperative parameters assessed were bone resection, monopolar instrument use, and reconstruction flap size. Bone resection was defined as positive if the bone was resected during the surgical procedure. Monopolar instrument use was defined as positive if a monopolar instrument was used during the surgical procedure to resect the tumor specimen. Reconstruction flap size was defined as small if a radial free forearm flap, scapula free flap, or soleus perforator flap was used and it was defined as large if an anterolateral thigh flap, fibula free flap, or latissimus dorsi flap was used for reconstruction. The histopathological postoperative parameters assessed were pathologic T-status (pT-status), grading, vascular invasion, and lymphatic invasion.

The IAR repetition numbers were defined according to the number of times IAR was performed in one patient during one surgery (zero if IAR was performed once, one if IAR was performed twice, and two if IAR was performed thrice).

Patients were categorized into two groups: those with IAR of microscopically involved resection margins in soft tissue to achieve definite microscopically uninvolved resection margins and those without IAR due to initially microscopically uninvolved resection margins in soft tissue. In addition, the patients were divided according to cT-status (above 2 and not above 2) and pT-status (above 2 and not above 2).

### Statistical analysis

Differences in clinicopathological baseline parameters between groups were analyzed using the chi-squared test or Fisher Freeman Halton test for categorical data, or the Mann–Whitney test for metric data. Differences in clinicopathological parameters (i.e., clinical preoperative, clinical intraoperative, and histopathological postoperative parameters) between groups were analyzed using the chi-squared test, Fisher’s exact test, or Fisher Freeman Halton test for categorical data, or the Mann–Whitney test for metric data. Associations between clinicopathological parameters and IAR were analyzed using univariable and multivariable logistic regression analysis. *P*-values < 0.05 were considered statistically significant. Statistical analysis was performed using SPSS Version 28 (SPSS, IBM, New York, USA).

## Results

### Study population

The study population consisted of 44 patients with IAR and 153 patients without IAR (Table 1). The groups did not differ in terms of sex, age, UICC stage, and definite resection margin distance (all *p* > 0.05). The groups differed with respect to adjuvant therapy (*p* = 0.008).

**Table 1:** Study population

**Variable**	**All patients (*n* = 197)**	**Without IAR (*n* = 153)**	**With IAR (*n* = 44)**	***p*-value**
***Sex*** (*n*)				
Male	107 (54.3%)	85 (55.6%)	22 (50.0%)	0.514
Female	90 (45.7%)	68 (44.4%)	22 (50.0%)	
***Age*** (years) (median (IQR))	63.0 (17.0)	64.0 (16.0)	61.5 (23.0)	0.581
***UICC stage*** (*n*)				
I	53 (26.9%)	44 (28.8%)	9 (20.5%)	0.123
II	36 (18.3%)	32 (20.9%)	4 (9.1%)	
III	37 (18.8%)	28 (18.3%)	9 (20.5%)	
IVa	58 (29.4%)	39 (25.5%)	19 (43.2%)	
IVb	13 (6.6%)	10 (6.5%)	3 (6.8%)	
IVc	0 (0.0%)	0 (0.0%)	0 (0.0%)	
***Therapy*** (*n*)				
surgery	103 (52.3%)	89 (58.2%)	14 (31.8%)	**0.008**
surgery + RTX	66 (33.5%)	44 (28.8%)	22 (50.0%)	
surgery + RCTX	28 (14.2%)	20 (13.1%)	8 (18.2%)	
***Definite resection margin distance*** (*n*)				
< 5mm	70 (35.5%)	51 (33.3%)	19 (43.2%)	0.229
≥ 5mm	127 (64.5%)	102 (66.7%)	25 (56.8%)	

Data presented as numbers (with percentage) (sex, UICC stage, therapy, definite resection margin distance) for categorical data and as median (with interquartile range) for metric data (age) separately for all patients and subdivided into patients without intraoperative additional resection (without IAR) and patients with intraoperative additional resection (with IAR); *p*-values corresponding to testing for differences between groups with chi-squared test (sex, therapy, definite resection margin distance), Freeman Halton test (UICC stage), and Mann Whitney test (age); significant *p*-values are bold

*Abbreviations: IAR* intraoperative additional resection, *UICC* Union for International Cancer Control, *RTX* radiotherapy, *RCTX* radiochemotherapy

### Predictive factors for intraoperative additional resection

**Fig. 1:**
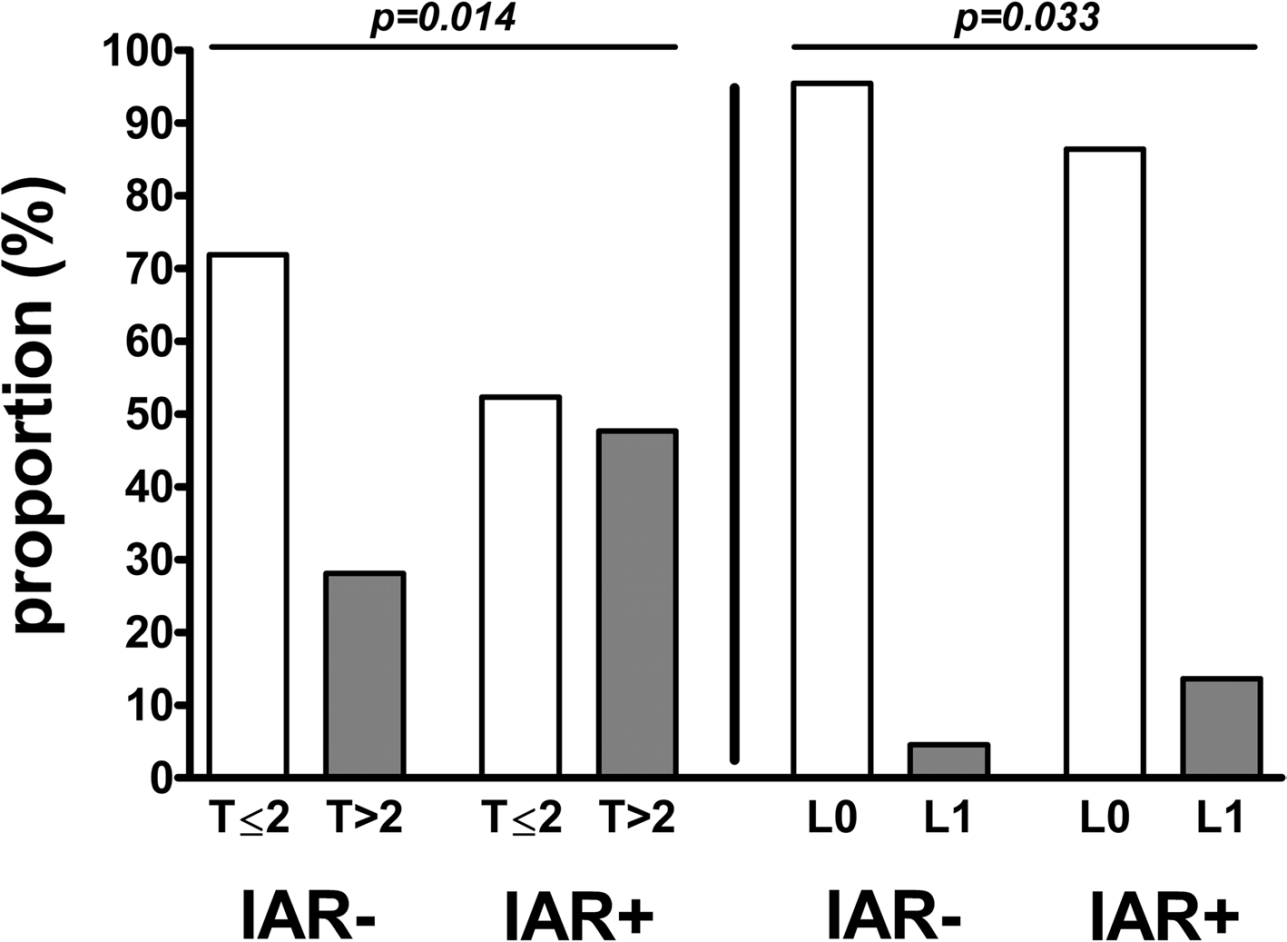
Comparison of groups with and without intraoperative additional resection. Data described as proportions (%) of patients without cT-status > 2 (T ≤ 2) and with cT-status > 2 (T > 2) and without lymphatic invasion (L0) and with lymphatic invasion (L1) separately described for patients without intraoperative additional resection (IAR-) and with intraoperative additional resection (IAR +); *p*-values corresponding to testing for differences between groups with chi-squared test (IAR- vs. IAR +); significant *p*-values are bold. *Abbreviations: T* ≤ *2*, cT-status ≤ 2; *T* > *2*, cT-status > 2; *L0*, lymphatic invasion negative; *L1*, lymphatic invasion positive; *IAR*, intraoperative additional resection

The groups did not differ in the assessed clinical preoperative and intraoperative parameters, such as tumor location, midline involvement, cT-status above 2, time interval between staging imaging and surgery, bone resection, monopolar instrument use, and reconstruction flap size (all *p* > 0.05) (Table 2). The groups also did not differ in two of the assessed histopathological postoperative parameters, namely, grading above 2 and vascular invasion (*p* = 0.833 and *p* = 1.000, respectively); however, they did differ in other histopathological postoperative parameters, namely, pT-status above 2 (with IAR: 47.7% vs. without IAR: 28.1%, *p* = 0.014) and lymphatic invasion (with IAR: 13.6% vs. without IAR: 4.6%, *p* = 0.033) (Table 2, Fig. 1).

**Table 2:** Comparison of clinical and histopathological parameters between groups

**Variable**	**All patients (*n* = 197)**	**Without IAR (*n* = 153)**	**With IAR (*n* = 44)**	***p*-value**
***Clinical preoperative parameters***				
***Tumor location*** (n)				
Tongue	61 (31.0%)	53 (34.6%)	8 (18.2%)	0.204
Floor of mouth	52 (26.4%)	41 (26.8%)	11 (25.0%)	
Mandible	28 (14.2%)	19 (12.4%)	9 (20.5%)	
Maxilla	18 (9.1%)	11 (7.2%)	7 (15.9%)	
Cheek	19 (9.6%)	14 (9.2%)	5 (11.4%)	
Soft palate	18 (9.1%)	14 (9.2%)	4 (9.1%)	
Hard palate	1 (0.5%)	1 (0.7%)	0 (0.0%)	
***Midline involvement*** (*n*)				
No	162 (82.2%)	124 (81.0%)	38 (86.4%)	0.416
Yes	35 (17.8%)	29 (19.0%)	6 (13.6%)	
***cT-status* > *2*** (*n*)				
No	147 (74.6%)	118 (77.1%)	29 (65.9%)	0.132
Yes	50 (25.4%)	35 (22.9%)	15 (34.1%)	
**Time SI-T** (days) (median (IQR))	11.0 (10.0)	11.0 (10.0)	13.5 (13.0)	0.329
***Clinical intraoperative parameters***				
**Bone resection** (*n*)				
No	90 (45.7%)	75 (49.0%)	15 (34.1%)	0.080
Yes	107 (54.3%)	78 (51.0%)	29 (65.9%)	
**Monopolar instrument use** (*n*)				
No	50 (25.4%)	42 (27.5%)	8 (18.2%)	0.213
Yes	147 (74.6%)	111 (72.5%)	36 (81.8%)	
**Reconstruction flap size** (*n*)				
Small	137 (69.5%)	108 (70.6%)	29 (65.9%)	0.552
Large	60 (30.5%)	45 (29.4%)	15 (34.1%)	
***Histopathological postoperative parameters***				
***pT-status* > *2*** (*n*)				
No	133 (67.5%)	110 (71.9%)	23 (52.3%)	**0.014**
Yes	64 (32.5%)	43 (28.1%)	21 (47.7%)	
***Grading* > *2*** (*n*)				
No	159 (80.7%)	123 (80.4%)	36 (81.8%)	0.833
Yes	38 (19.3%)	30 (19.6%)	8 (18.2%)	
***Vascular invasion*** (*n*)				
No	193 (98.0%)	150 (98.0%)	43 (97.7%)	1.000
Yes	4 (2.0%)	3 (2.0%)	1 (2.3%)	
***Lymphatic invasion*** (*n*)				
No	184 (93.4%)	146 (95.4%)	38 (86.4%)	**0.033**
Yes	13 (6.6%)	7 (4.6%)	6 (13.6%)	

Data presented as numbers (with percentage) (tumor location, midline involvement, cT-status, bone resection, monopolar instrument use, reconstruction flap size, pT-status, grading, vascular invasion, lymphatic invasion) for categorical data and as median (with interquartile range) for metric data (time interval SI-T) separately for all patients and subdivided into patients without intraoperative additional resection (without IAR) and patients with intraoperative additional resection (with IAR); reconstruction flap size small: radial free forearm flap, scapula free flap, soleus perforator flap; reconstruction flap size large: anterolateral thigh flap, fibula free flap, latissimus dorsi flap; *p*-values corresponding to testing for differences between groups with chi-squared test (midline involvement, cT-status, bone resection, monopolar instrument use, reconstruction flap size, pT-status, grading, lymphatic invasion), Freeman Halton test (tumor location), Fisher’s exact test (vascular invasion), and Mann–Whitney test (time interval SI-T); significant *p*-values are bold

*Abbreviations: AR* intraoperative additional resection, *cT-status* clinical T-status, *SI-T* staging imaging to therapy interval, *pT-status* histopathologic T-status

The univariable analysis results indicated that patients with a pT-status above 2 were more likely to have undergone IAR (odds ratio [OR] 2.336 [confidence interval [CI] 1.173–4.650], *p* = 0.016) and that patients with lymphatic invasion were more likely to have undergone IAR (OR 3.293 [CI 1.046–10.373], *p* = 0.042) (Table 3). The multivariable analysis results indicated that only patients with a pT-status above 2 were more likely to have undergone IAR (OR 2.062 [CI 1.008–4.221], *p* = 0.048) (Table 3).

**Table 3:** Regression analysis

**Parameter**	**Parameter comparison**	**Univariable testing**		**Multivariable testing**	
		Odds ratio (95% CI)	*p*-value	Odds ratio (95% CI)	*p*-value
pT-status > 2	no vs yes	2.336 (1.173—4.650)	**0.016**	2.062 (1.008—4.221)	**0.048**
Lymphatic invasion	no vs yes	3.293 (1.046—10.373)	**0.042**	2.394 (0.724—7.919)	0.153

Odds ratios (with confidence interval) and* p*-values corresponding to univariable and multivariable regression analysis for intraoperative additional resection; significant *p*-values are bold

*Abbreviations: pT-status* histopathologic T-status, *CI* confidence interval

### Repetition numbers of intraoperative additional resection

**Fig. 2:**
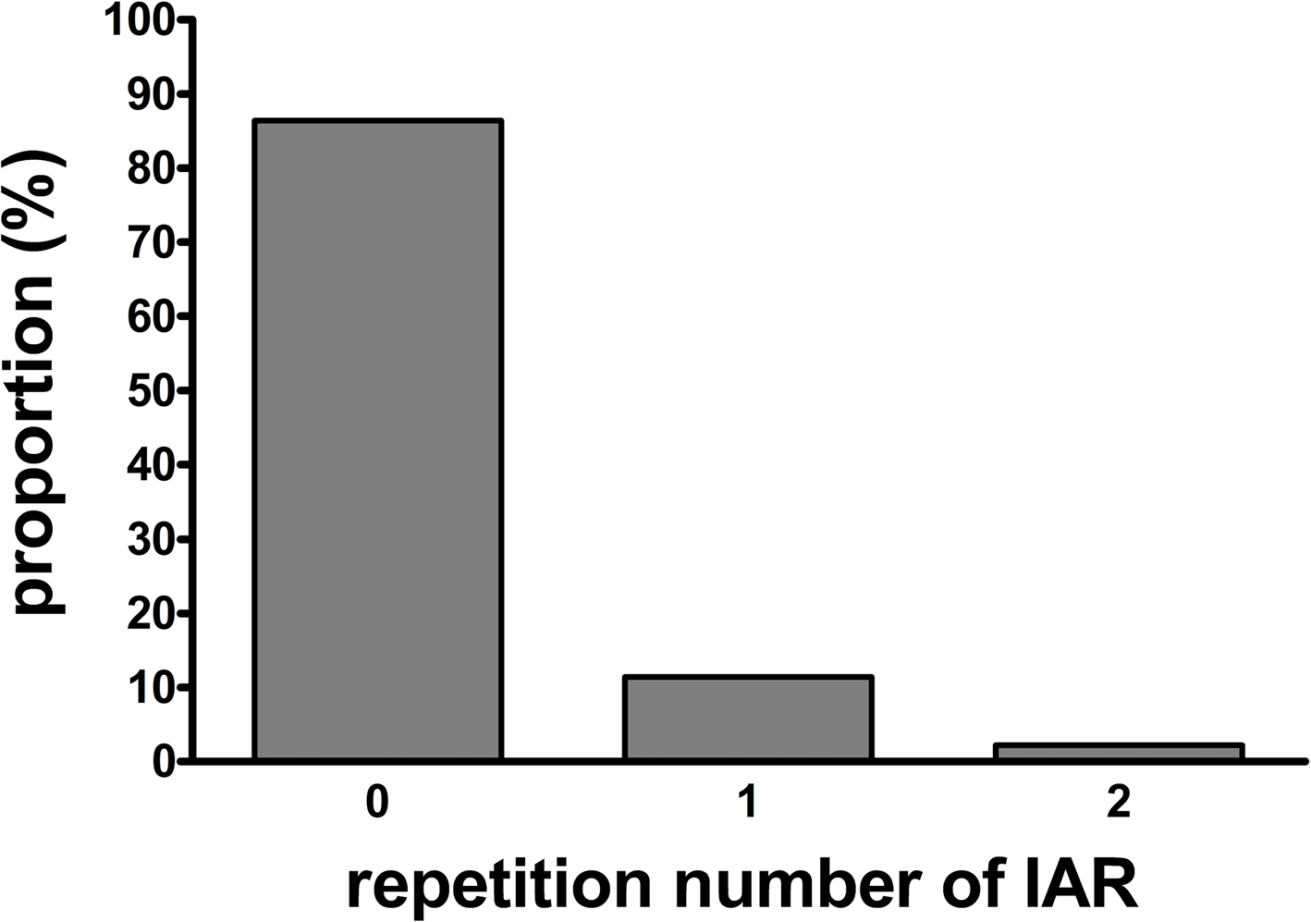
Repetition numbers of intraoperative additional resection. Data described as proportions (%) of repetition numbers of intraoperative additional resection described for patients with intraoperative additional resection

In terms of the number of times IAR was repeated in the patients who underwent IAR, a value of zero was assigned to 38 patients (86.4%), a value of one was assigned to 5 patients (11.4%), and a value of two was assigned to one patient (2.3%) (Fig. 2). No associations were found between the values and the clinical preoperative, clinical intraoperative, or histopathological postoperative parameters (all *p* > 0.05) (Table 4).

**Table 4:** Repetition numbers of intraoperative additional resection

**Variable**	**0**	**1**	**2**	***p*-value**
***Clinical preoperative parameters***				
***Tumor location*** (*n*)				
Tongue	8 (100.0%)	0 (0.0%)	0 (0.0%)	0.194
Floor of mouth	10 (90.9%)	1 (9.1%)	0 (0.0%)	
Mandible	8 (88.9%)	1 (11.1%)	0 (0.0%)	
Maxilla	5 (71.4%)	2 (28.6%)	0 (0.0%)	
Cheek	5 (100.0%)	0 (0.0%)	0 (0.0%)	
Soft palate	2 (50.0%)	1 (25.0%)	1 (25.0%)	
Hard palate	0 (0.0%)	0 (0.0%)	0 (0.0%)	
***Midline involvement*** (n)				
No	33 (86.8%)	4 (10.5%)	1 (2.6%)	0.609
Yes	5 (83.3%)	1 (16.7%)	0 (0.0%)	
***cT-status* > *2*** (*n*)				
No	25 (86.2%)	3 (10.3%)	1 (3.4%)	1.000
Yes	13 (86.7%)	2 (13.3%)	0 (0.0%)	
***Clinical intraoperative parameters***				
**Bone resection** (*n*)				
No	14 (93.3%)	1 (6.7%)	0 (0.0)	0.764
Yes	24 (82.8%)	4 (13.8%)	1 (3.4%)	
**Monopolar instrument use** (*n*)				
No	6 (75.0%)	1 (12.5%)	1 (12.5%)	0.212
Yes	32 (88.9%)	4 (11.1%)	0 (0.0%)	
**Reconstruction flap size** (*n*)				
Small	26 (89.7%)	3 (10.3%)	0 (0.0%)	0.554
Large	12 (80.0%)	2 (13.3%)	1 (6.7%)	
***Histopathological postoperative parameters***				
***pT-status* > *2*** (*n*)				
No	20 (87.0%)	3 (13.0%)	0 (0.0%)	0.824
Yes	18 (85.7%)	2 (9.5%)	1 (4.8%)	
***Grading* > *2*** (*n*)				
No	30 (83.3%)	5 (13.9%)	1 (2.8%)	0.644
Yes	8 (100.0)	0 (0.0%)	0 (0.0%)	
***Vascular invasion*** (*n*)				
No	37 (86.0%)	5 (11.6%)	1 (2.3%)	1.000
Yes	1 (100.0%)	0 (0.0%)	0 (0.0%)	
***Lymphatic invasion*** (*n*)				
No	33 (86.8%)	4 (10.5%)	1 (2.6%)	0.609
Yes	5 (83.3%)	1 (16.7%)	0 (0.0%)	

Data presented as numbers (with percentage) (tumor location, midline involvement, cT-status, bone resection, monopolar instrument use, reconstruction flap size, pT-status, grading, vascular invasion, lymphatic invasion) for patients with intraoperative additional resection (*n* = 44); reconstruction flap size small: radial free forearm flap, scapula free flap, soleus perforator flap; reconstruction flap size large: anterolateral thigh flap, fibula free flap, latissimus dorsi flap; *p*-values corresponding to testing for differences between groups with Freeman Halton test

*Abbreviations*: *cT-status* clinical T-status, *pT-status* histopathologic T-status

## Discussion

This study investigated whether predictive factors for IAR in soft tissue exist in terms of clinical preoperative and intraoperative parameters and the number of times IAR is performed in soft tissue varies and is dependent on these parameters in the surgical treatment of OSCC.

In cases of OSCC, given that resection with IAR to clear initially microscopically involved resection margins is associated with lower local control than resection without IAR, probably due to tumor cut through with dissemination of tumor cells and/or a more aggressive tumor biology both of which are indicated by initially microscopically involved resection margins, there is a need to avoid IAR to reduce tumor recurrence and improve patient outcome [4, 8–12, 15, 18–20]. Of note, although other factors also influence OSCC recurrence, resection margin status or avoidance of IAR is the only factor that can be influenced by the surgeon [3, 6, 8]. However, clinical assessment of the tumor margin to adequately select the initial macroscopic resection margin distance based on imaging, visual inspection, and palpation is inaccurate [3, 8, 11, 21]. Therefore, apart from improvements in imaging that will allow preoperative determination of tumor margins or the development of methods that will allow intraoperative visualization of tumor margins, one approach that could be applied to avoid IAR is to increase the initial macroscopic resection margin distance beyond the commonly used 1 cm [8, 11, 12, 16, 22, 23].

In terms of clinical implications, information on predictive factors for IAR and IAR repetition numbers could be used to determine when and how to increase the initial macroscopic resection margin distance. However, the literature lacks sufficient information on both aspects, as previous studies have not considered potentially predictive factors for IAR nor examined IAR repetition numbers [3, 9].

This study only included patients with definite microscopically uninvolved resection margins, a cohort that represented the general possibility of complete tumor resection unrestricted by infiltration of vital structures (e.g., skull base) or by limitations for further surgical intervention (e.g., reduced patient condition), with a group of patients who had not undergone IAR as the reference standard for comparison with a group of patients who had undergone IAR [1, 10, 16, 24]. Interestingly, patients with and without IAR did not differ in terms of the definite microscopic resection margin distance (i.e., ≥ 5 mm and < 5 mm), suggesting that not only are definite microscopically uninvolved resection margins achievable in patients with IAR but also comparable definite microscopic resection margin distances [5]. Patients with postoperative additional resection were excluded, as relocation of initially microscopically involved resection margins is likely to be more difficult postoperatively due to postoperative tissue retraction and altered anatomy, which particularly affects IAR repetition numbers [8, 12, 13, 24].

In this study, only the histopathological postoperative parameter of a pT-status above 2 was identified as a predictive factor for IAR. Lymphatic invasion was not associated with IAR in the multivariable analysis. In contrast, tumor location, midline involvement, cT-status above 2, and time interval between staging imaging and surgery did not differ between the groups. This may be unexpected given that tumor location affects accessibility within the oral cavity, larger tumors are more likely to exhibit greater three-dimensional anatomical complexity and irregular extension, and discrepancies in tumor dimensions between staging imaging and the day of surgery increase over time; all of these factors presumably contribute to a more challenging tumor resection procedure and increase the chance of IAR [12, 21, 24–27]. In this context, for example, one study showed that tumor location of OSCC in the cheek was associated with the poorest local control compared with other sites in the oral cavity [28]. In addition, bone resection, monopolar instrument use, and reconstruction flap size did not differ between the groups. This may be unexpected given that bone resection reflects that an extensive resection was required, resection with a monopolar instrument could lead to greater difficulty in assessing the resection margin because of tissue retraction, and a larger reconstruction flap is typically indicative of a more aggressive surgery due to the use of a greater quantity of tissue for defect coverage; all of these factors presumably influence the need for IAR [13, 29, 30]. The histopathological parameters grading above 2 and vascular invasion also did not differ between the groups, although both are thought to indicate tumor aggressiveness and tumor extension beyond the visible tumor margins, which likely lead to inadequate selection of the initial macroscopic resection margin distance and subsequent IAR [6, 13, 21]. In general, the findings of this study are consistent with observations that tumor location, cT-status, grading, vascular invasion, and lymphatic invasion do not differ between patients with and without IAR [3, 9]. The identification of pT-status above 2 rather than cT-status above 2 as a predictive factor for IAR could be due to the more accurate representation of tumor dimensions by pT-status than by cT-status [31].

The identification of pT-status above 2 as a predictive factor for IAR could be related to the fact that larger tumors are more likely to be three-dimensionally complex and less accessible in the oral cavity, making tumor resection more difficult and resulting in IAR [21, 26, 32]. However, for the purpose of identifying a reliable indicator for increase of the initial macroscopic resection margin distance, no preoperative or intraoperative predictive factor could be identified since pT-status is first available postoperatively after complete tumor resection, and preoperative biopsies do not share the histopathological parameters of the postoperative resection specimen [33].

In this study, an evaluation of IAR repetition numbers showed that additional resection was performed only once in the majority of cases (> 85.0%). Interestingly, in one other study, increasing the initial macroscopic resection margin distance from 1 to 1.5 cm resulted in a lower number of microscopically involved resection margins in the surgical treatment of OSCC in the tongue [34]. Hence, increasing the initial macroscopic resection margin distance to 1.5 cm might be sufficient for avoiding IAR in the majority of cases in the surgical treatment of OSCC.

The limitations of this study include the small number of patients, the lack of data for perineural invasion, and the use of three different UICC classifications for staging the patients, which particularly hampered the comparison of patients’ pT-status [35]. However, the groups did not differ for UICC classification (6th and 7th vs. 8th edition; chi-squared test *p* > 0.05). In addition, the lack of data on cancer immunology, e.g., tumor cell antigen expression, which relates to the interaction between tumor cells and immune cells and consequently plays a central role in tumor invasion, should be considered a limitation of the study [36].

Our findings show that none of the preoperative or intraoperative parameters examined in this study have potential as predictive factors for IAR, which can be used to determine when the initial macroscopic resection margin distance should be increased beyond the commonly used 1 to 1.5 cm to avoid IAR in soft tissue in the surgical resection of OSCC. However, in the absence of reliable predictive factors, it cannot be recommended to generally increase the initial macroscopic resection margin distance, because this may adversely affect the balance between adequate tumor resection and satisfactory organ preservation [4, 7, 8, 22]. Therefore, with regard to preserving the functionally important structures in the oral cavity, an individualized approach with an increase of the initial macroscopic resection margin distance only if necessary is needed [4, 16].

In the context of the limited accuracy of preoperative imaging, visual inspection, and palpation, this study underscores the need for further improvement in intraoperative tumor margin visualization [8, 16, 23].

## Conclusions

Increasing the initial macroscopic resection margin distance beyond the tumor margin from the commonly used 1 to 1.5 cm could avoid the need for IAR in soft tissue in most cases in the surgical treatment of OSCC. However, only the histopathological postoperative parameter of a pT-status above 2, and no clinical preoperative or intraoperative available parameter, was identified as a predictive factor for IAR. This underscores the need for improvement of preoperative imaging and intraoperative tumor visualization to adequately determine tumor borders and select an appropriate initial macroscopic resection margin distance beyond the tumor margin to avoid IAR and thus improve local control in the treatment of OSCC.

## Data Availability

The datasets analyzed during the current study are available from the corresponding author upon reasonable request.

## Abbreviations

IAR: Intraoperative additional resection
OSCC: Oral squamous cell carcinoma
cT-status: Clinical T-status
pT-status: Pathologic T-status
